# Compliance with the 2018 World Cancer Research Fund/American Institute for Cancer Research Cancer Prevention Recommendations and Prostate Cancer

**DOI:** 10.3390/nu12030768

**Published:** 2020-03-14

**Authors:** Olmedo-Requena Rocío, Lozano-Lorca Macarena, Salcedo-Bellido Inmaculada, Jiménez-Pacheco Antonio, Vázquez-Alonso Fernando, García-Caballos Marta, Sánchez María-José, Jiménez-Moleón José-Juan

**Affiliations:** 1 Department of Preventive Medicine and Public Health, University of Granada, 18016 Granada, Spain; macarenalozano@ugr.es (L.-L.M.); isalcedo@ugr.es (S.-B.I.); mariajose.sanchez.easp@juntadeandalucia.es (S.M.-J.); jjmoleon@ugr.es (J.-M.J.-J.); 2 Consortium for Biomedical Research in Epidemiology and Public Health (CIBERESP), 28029 Madrid, Spain; 3 Instituto de Investigación Biosanitaria (ibs.GRANADA), 18014 Granada, Spain; 4 Urology Department, San Cecilio University Hospital, 18016 Granada, Spain; anjipa29@hotmail.com; 5 Urology Department, Virgen de las Nieves University Hospital, 18014 Granada, Spain; fvazquezalonso@gmail.com; 6 Cartuja Primary Health Care Centre, Distrito Sanitario Granada-Metropolitano, 18013 Granada, Spain; martagc81@gmail.com; 7 Andalusian School of Public Health (EASP), 18011 Granada, Spain

**Keywords:** prostate cancer, WCRF/AICR 2018 recommendations, nutrition-based guidelines, case-control study

## Abstract

The etiology of prostate cancer (PCa) remains largely unknown. Compliance with the 2018 World Cancer Research Fund/American Institute for Cancer Research (WCRC/AICR) cancer prevention recommendations and its relationship to PCa was evaluated. A total of 398 incident PCa cases and 302 controls were included. The selection criteria for both cases and controls were: (i) age between 40–80 years; and (ii) residence in the coverage area of the reference hospitals for 6 months or more prior to recruitment. A score to measure the compliance with the recommendations of 2018 WCRC/AICR criteria was built. The level of compliance was used as a continuous variable and categorized in terciles. The aggressiveness of PCa was determined according to the ISUP classification. Adjusted odds ratios (aOR) and their 95% confidence intervals (95% CI) were estimated using multivariable logistic regression models. A slight protective tendency was observed between the level of compliance with the preventive recommendations and PCa risk, aOR = 0.81 (95% CI 0.69–0.96) for the total cases of PCa. This association also was observed when the aggressiveness was considered. In addition, limiting consumption of “fast foods”, sugar-sweetened drinks, and alcohol were independently associated with lower risk of PCa.

## 1. Introduction

Prostate cancer (PCa) is the most common cancer in men, and has the highest incidence and the third highest mortality, after lung and colorectal cancer, in Europe [1]. In recent years, the incidence of PCa has become widespread in all countries [2], with around 450,000 estimated new cases per year [3]. This increase may be related to population aging and new exposures, or higher levels of exposure, to environmental risk factors, together with the massive use of screening for prostate-specific antigen (PSA) and diagnostic techniques [4].

Despite its considerable impact, the etiology of PCa remains largely unknown, and only the following risk factors are considered as well-established: age, ethnicity [2], hereditary factors, and genomic [5,6,7]. All of these are non-modifiable factors. Other factors related to adherence to unhealthy lifestyles have been proposed, such as poor quality of the diet, physical inactivity or sedentariness, and obesity, but their individual roles in the etiology of PCa remains unclear [8,9,10]. Regardless of the relationship between diet, physical activity, and body weight and PCa, these factors have been associated with other types of cancer such as breast or colorectal cancer [11,12]. Therefore, it could be interesting to explore the relationship between the combination of these three factors and the risk of PCa.

In 2007, the World Cancer Research Fund (WCRF) and the American Institute for Cancer Research (AICR) published the Second Expert Report for cancer prevention, replacing the First Expert Report conducted in 1997 [13,14]. This report included a total of 10 recommendations related to diet, physical activity, and body fatness for the prevention of cancer. However, these recommendations did not establish a quantitative standard system to manage them, this complicates the comparison between studies. In 2018, the Third Expert Report was published (Diet, Nutrition, Physical Activity, and Cancer: A Global Perspective) [15], and one year later a standardized scoring system was developed and published [16].

Since 2007, several studies have evaluated the association of the recommendations of the Second Expert Report and cancer mortality [17], as well as the risk of developing cancer [18]; especially with breast cancer, colorectal cancer, head and neck cancer, pancreatic cancer, lymphocytic leukemia, and PCa [19,20,21,22,23,24,25,26,27,28,29,30,31]. However, as we have commented previously, the comparison of the results is not easy because of the absence of standard criteria. Furthermore, the association between 2018 WCRF/AICR Cancer Prevention Recommendations and PCa has not yet been studied. In addition, PCa may not be a single pathology, and its behavior may be different depending on the degree of aggressiveness. Overdiagnosis poses a fundamental problem, especially in low aggressive cases [32]. Therefore, it is necessary to evaluate the new standardize recommendations and their association with PCa taking into account the grade of aggressiveness. The aim of the present study is to evaluate the effect of compliance with the 2018 nutrition-based guidelines of the WCRF/AICR cancer prevention recommendations on the risk of PCa and its association with the aggressiveness at diagnosis.

## 2. Materials and Methods

### 2.1. Study Design and Setting

A population-based case-control study was developed to evaluate the association between lifestyles and PCa among Spanish men (CAPLIFE study). It was carried out at in two main University Hospitals in Granada (Spain): Virgen de las Nieves and San Cecilio Hospitals and their catchment area. They belong to Metropolitan Granada District, which covers more than 645,000 inhabitants. All those PCa cases that belonged to the aforementioned hospitals were invited to participate by their urologist after being diagnosed of PCa. Study participants were enrolled from May 2017 to December 2019.

Ethical Approval for this study was provided by the Ethics Committee of Biomedical Research of Andalusia in March 2017. All men included in the study were fully informed about the study objectives and they signed a written informed consent before their voluntary participation. Confidentiality of data is secured by removing personal identifiers in the dataset.

### 2.2. Participants

Eligible cases were required to meet the following criteria: (1) new diagnosis of PCa (International Classification of Diseases 10th Revision [ICD-10]: C61) [33] with histological confirmation. We have only included incident cases before starting any type of treatment for PCa; (2) age between 40 and 80 years; (3) residence in the coverage area of the reference hospitals for 6 months or more prior to recruitment. The same criteria were used for controls, except diagnosis of PCa. The incident cases were selected from the Urology Department of the participating hospitals, using the Pathological Anatomy listings and checking the new positive biopsies for PCa. Controls were randomly selected from the population lists assigned to general practitioners of the primary care centers located in the catchment area of the hospitals from which the cases were selected (Granada-Metropolitan Sanitary District). The controls were matched to cases by age, with a maximum age difference of 5 years, based on information from the Granada Cancer Registry, a population-based cancer registry with data quality certified by the International Agency for Research on Cancer (http://cancergranada.org/es/index.cfm).

### 2.3. Data Sources and Variables

Data were collected between 2017 and 2019 by face-to-face interviews conducted by trained interviewers using a structured computerized epidemiological questionnaire. The same interviewers surveyed cases and controls. Information on the following background variables, for both cases and controls, was obtained: sociodemographic data (age, education level, occupation, and marital status), lifestyles (smoking status, alcohol consumption, physical activity, sleeps habits, and diet), and personal/family medical history, including first-degree family history of PCa in father and/or brothers, among other variables. Waist and hip circumference were measured at the interview, while height and weight one year prior to diagnosis were self-reported, and body mass index (BMI) calculated. If there were any missing data, it was recovered by reviewing the clinical record or via telephone contact.

The physical activity information was collected using the International Physical Activity Questionnaire (IPAQ), validated for the Spanish population [34]. In addition, subjects were provided with a semi-quantitative food frequency questionnaire (FFQ); also previously validated for the Spanish population [35] which included 134 foods, specifying the portion size for each of them, referring to the 12 months prior to diagnosis. Nutrient intakes were estimated using Spanish food composition tables [36]. In addition, this study included only subjects with plausible energy intakes. An intake of less than 800 kcal/day and more than 4000 kcal/day were considered as implausible extreme energy intakes [37].

#### 2.3.1. WCRF/AICR Score Construction

The 2018 WCRF/AICR Cancer Prevention Recommendations includes a total of ten recommendations: (1) body fatness, (2) physical activity, (3) consumption of whole grains, vegetables, fruit, and beans, (4) fast food and other processed foods, (5) red and processed meat, (6) sugar-sweetened drinks, and (7) alcohol, (8) dietary supplements, (9) breastfeeding, and (10) cancer survivors’ recommendations. In this study, we omitted the latest three recommendations. The reasons for avoiding the three last recommendations were the following: (i) The use of dietary supplements for cancer prevention and choice to consume nutrients through food alone is largely addressed through the other five dietary recommendations, as AICR refers in the article on the operationalizing of the score [16]; and (ii) the specific recommendation for cancer survivors and breastfeeding are not applicable to our population. Table 1 shows the goals for the ten recommendations and their operationalization.

Briefly, the method of estimating the score according to the standardized scoring system for 2018 WCRF/AICR cancer prevention recommendations [16] is based on the following criteria: 1 point was assigned when the recommendation was met, 0.5 points when it was partially met, and 0 points when not met. When the recommendation was composed of two subitems, such as body fatness and consumption of whole grains, vegetables, fruit, and beans recommendation, the scoring weight is divided equally between both to retain a total of one point (0.5, 0.25, and 0 points for meeting, partially meeting, and not meeting each subitem, respectively).

In particular, for the recommendation on “fast foods” we selected all ultra-processed foods–according to the NOVA classification [38]–available in the FFQ. After excluding those items already included in other recommendations (i.e., processed meats, sweetened drinks, and alcoholic beverages), we calculated the proportion of calorie intake (kcal/day) derived from ultra-processed foods (group 4 NOVA classification) with respect to the total caloric intake per day. The ratio of fast food caloric intake divided by total caloric intake was finally broken down into terciles according to its distribution in the control group.

The score of each recommendation was added to obtain the total score, which ranged from a minimum value of 0 to a maximum value of 7 points, with higher values indicating high compliance with the cancer prevention recommendations. From the score obtained for the control group, the cut-off points of the terciles were established and applied to both groups to define the level of compliance: (i) Tercile 1: minimal compliance with the recommendations; (ii) Tercile 2: intermediate compliance and; (iii) Tercile 3: minimum compliance.

#### 2.3.2. Measurement of Tumor Aggressiveness

The Gleason score was collected from the pathological report of each participant. It assigns two grades for each patient: a primary grade is given to describe the cells that make up the largest area of the tumor and a secondary grade is given to describe the degree of differentiation of the cells of the next largest area. Both grades use values from 1 to 5: 1 for a high degree of differentiation and 5 for a minimal degree of differentiation [39]. The aggressiveness of the tumor was determined according to the classification of the International Society of Urological Pathology (ISUP) [40], which establishes the existence of five grade groups that are: (i) ISUP 1 (Gleason 3 + 3); (ii) ISUP 2 (Gleason 3 + 4); (iii) ISUP 3 (Gleason 4 + 3); (iv) ISUP 4 (Gleason 8); and (v) ISUP 5 (Gleason > 8). From this, two categories of aggressiveness were constructed: low aggressiveness (ISUP 1, 2) and high aggressiveness (ISUP 3, 4, 5) [41].

### 2.4. Statistical Analysis

Characteristics of the participants were examined using means and standard deviations (SD) for continuous variables and percentages for categorical variables. These characteristics were described for case and control groups as well as 2018 WCRF/AICR terciles for the control group. Chi-squared tests were used to evaluate the level of significance of the differences observed in categorical variables, and Student’s t-tests or one-way ANOVA for continuous variables.

Multivariable logistic regression models were used to estimate odds ratios (OR) and 95% confidence intervals (95% CI) for the association between the 2018 WCRF/AICR recommendations and PCa. The 2018 WCRF/AICR score was analyzed as a continuous variable (one-unit increment) and as a categorical variable through the terciles. The first tercile was used as the reference category (minimum compliance with the cancer prevention recommendations). We used the information from previous studies and directed acyclic graph (DAG) to identify potential confounders; thus, epidemiological and statistical criteria were used to construct the models. An adjusted model was constructed including as covariates: age, educational level, smoking status, primary family history of PCa, and total energy intake. In addition, analyses were also conducted stratifying by aggressiveness: low aggressiveness and high aggressiveness. We estimated the individual association of each component of the 2018 WCRF/AICR score with PCa risk, after adjustment for all other components of the score and the aforementioned potential confounders. For this analysis, participants without information on anthropometric measures, physical activity, or dietary information were excluded. People with implausible energy intake values were also excluded for the analysis. All statistical tests were two-sided and statistical significance was set at *p* < 0.05. Statistical analyses were performed using statistical program Stata v.15 (Stata Corp., College Station, TX, USA, 2017).

## 3. Results

**Figure 1:**
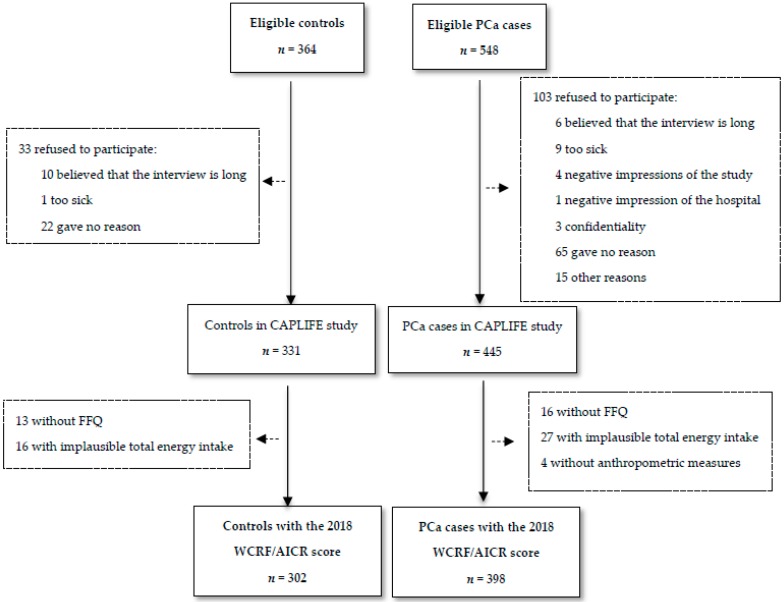
Flow-chart CAPLIFE study.

Figure 1 shows the flow-chart diagram for the participants in the CAPLIFE study until December 2019. A total of 398 PCa cases and 302 controls had complete information for the 2018 WCRF/AICR score and a plausible total energy intake. There were no statistically significant differences between controls with and without information on the score and PCa cases with and without the score, except for the educational level in PCa cases (Supplementary Table S1).

Distribution characteristics between cases and controls are shown in Table 2. Compared with controls, PCa cases were slightly older, 67.7 (SD 7.4) vs. 65.3 (8.2) years, they had a higher alcohol consumption (16.8 for cases vs. 10.3% for controls) and a higher energy intake, 2511.8 (SD 705.7) vs. 2438.1 (SD 617.6) kcal/day. More than three quarters of the PCa cases were of low aggressiveness (77.1%) cases with an ISUP of 1 or 2. In terms of compliance with the 2018 WCRF/AICR score, this was slightly lower in the PCa cases than in controls, 3.27 (DS 0.93) vs. 3.42 (DS 1.01) points, respectively. The highest percentage of PCa cases (40.2%) had a score >2 and ≤ 3 points, while 34.8% controls had a score >3 and ≤ 4 points.

The distribution of controls’ characteristics according to terciles of compliance with the score is provided in Table 3. Those participants with the highest compliance vs. subjects with the lowest compliance with the 2018 WCRF/AICR recommendations were older, had a lower energy intake, and had lower alcohol consumption (3.3% with high alcohol consumption in controls with the highest compliance of the recommendations vs. 19.8% in controls with the lowest compliance; *p* < 0.01).

When we analyzed the association with recommendation compliance, a slight protective relationship was observed between a higher compliance with the 2018 WCRF/AICR recommendations and PCa when this variable was analyzed as continuous (Table 4). For each unit of increase in the score, the risk of PCa was reduced by 19%, aOR = 0.81 (95% CI 0.69–0.96) (*p*-value = 0.02). Its protective association is maintained when the 2018 WCRF/AICR score is considered as an ordinal variable in terciles, although without reaching statistical significance. The results are similar when we stratify by aggressiveness, aOR = 0.79 (95% CI 0.66–0.95) and aOR = 0.86 (95% CI 0.69–1.06) for each unit of increase in the score for low and high aggressiveness, respectively.

The mutually aOR for the individual components of the 2018 WCRF/AICR score and PCa are shown in Table 5. Three of the components of the score were independently associated with a lower risk of PCa: fast food and other processed foods, sugar-sweetened drinks, and alcohol consumption. Lower consumption of “fast foods’ and other processed foods high in fat, starches or sugars, and sugar-sweetened drinks were associated with a lower risk of PCa, aOR = 0.63 (95% CI 0.42–0.64) and aOR = 0.30 (95% CI 0.12–0.77), respectively (*p*-trend < 0.05). Also, not consuming alcohol showed a protective association, aOR = 0.49 (95% CI 0.27–0.91) for men who met with the recommendations (*p*-trend < 0.05). Results according to aggressiveness are detailed in Supplementary Table S2 and similar results were obtained for all of the individual items; although these results should be taken with caution because of the sample size.

## 4. Discussion

This case-control study explores the association between the 2018 WCRF/AICR Cancer Prevention Recommendations and the risk of PCa, being one of the first studies in exploring this issue. A slight protective association was found between each one-unit increase of the 2018 WCRF/AICR score and the risk of PCa, especially for low aggressiveness PCa. In addition, three components of the 2018 WCRF/AICR score were independently associated with a lower risk of PCa: (i) “Fast foods” and other processed foods high in fat, starches or sugars; (ii) sugar-sweetened drinks; and (iii) alcohol.

If we compare the 2018 WCRF/AICR score with the previous recommendations, six of them partially coincide, while the following four recommendations have changed substantially: physical activity, alcohol intake, “fast foods” and other processed foods, and sugar-sweetened drinks [15]. In particular, we have found an association for three of the four changed recommendations. These changes permit a better definition of the recommendations and their characteristics, reducing the chances of misclassification. The physical activity recommendation of ≥30 min/day without indicating intensity was changed and subsequently considered only moderate-vigorous physical activity. In terms of alcohol consumption, the new recommendation supports a consumption equal to zero, instead of a daily intake of no more than two drinks for men in the 2007 WCRF/AICR recommendations. This recommendation is similar to the advice included in the European Code against Cancer [42]. Regarding foods and drinks that promote weight gain, the previous recommendation of 2007 is divided in two new recommendations: consumption of “fast foods” and other processed foods, and sugar-sweetened drinks.

To date the association between compliance with the 2018 WCRF/AICR recommendations and PCa has not been evaluated, but it has already been explored for the 2007 WCRF/AICR recommendations. The studies that use the previous recommendations do not find an association between these recommendations and PCa when the aggressiveness of the tumor is not considered [21,22,23,29,31]. The absence of predefined cut-off points for the different items of the 2007 recommendations might also have contributed to these negative results.

By stratifying for aggressiveness, a protective association between the cancer prevention recommendations and low aggressiveness PCa was observed; a finding not previously found by other researchers. For cases with a tumor with high aggressiveness, we also observed a protective effect, although the results do not reach the statistical significance. This may be due to the small sample size for high aggressiveness PCa in our study. In fact, other studies find similar results, MCC-Spain [22], where an inverse association was found for Gleason score ≥ 7 cases (aOR = 0.85, 95% CI 0.76–0.96, per 1-point increment in the WCRF/AICR score). A similar association (aOR = 0.87, 95% CI 0.79–0.96, per 1-point increment in the WCRF/AICR score) was observed in the North Carolina-Louisiana Prostate Cancer Project [30].

Regarding limiting consumption of “fast foods” and other processed foods high in fat, starches or sugars, a recent cohort study, published in 2018, studied the association between “fast foods” and processed food and PCa risk [43]. This study found associations between ultra-processed foods and cancer in general, and breast cancer in particular; for PCa, no relation with consumption of fast food was observed, however only 281 PCa cases were analyzed.

For sugar-sweetened drinks and PCa, the results are also limited. According to a meta-analysis carried out with four observational studies conducted before 2012, the results are highly heterogeneous and they did not find an association between carbonated beverages and PCa [44]. In our study, we have considered both carbonated beverages and industrial sugar juices, and this difference could explain the inconsistent results. The French NutriNet-Santé prospective cohort (2009-2017) suggests a relationship between drink consumption and the total risk of cancer, producing an increase in the total risk of cancer of 18% per 100 mL per day [45]. In this cohort study, a higher risk is also observed for PCa, although it did not reach statistical significance because of a low sample size (291 PCa cases). Data on alcohol consumption and PCa is far more consistent, and a recent meta-analysis points to positive association for PCa; this risk starts even with a low volume consumption of alcohol (from 1.3 g per day) [46].

We found that PCa cases without a WCRF/AICR score had a lower educational level (Supplementary Table S1), as subjects with a lower educational level tend to have a lower compliance with recommendations, this would be underestimating the findings found. We may have been limited by the small sample size and lack of statistical power to detect significant associations, especially among high aggressiveness PCa cases. Finally, although we adjusted for a range of potential confounders which were associated with both the score and PCa risk, we cannot rule out the possibility of residual confounding.

Regarding advantages of our study, we have to point out the use of a previously validated FFQ for the Spanish population, including regional products [35]. In order to avoid possible changes in dietary habits after cancer diagnosis, we have only included incident cases before starting any type of treatment for PCa. In addition, the approach using the 2018 WCRF/AICR recommendations permits the evaluation of diet as a whole, accounting for possible synergistic effects between nutrients and foods on cancer risk, and it also incorporates physical activity and body fatness; hence being an indicator of an overall healthy lifestyle. As an additional strength of our study, the 2018 WCRF/AICR recommendations have been based on a standardized scoring system [16], allowing the construction of comparable and solid cut-off points for each recommendation, a problem found in the previous applications of this score. It is also noteworthy, that most of the participants of the CAPLIFE study had detailed information about the diet and plausible energy intake which allowed the construction of the score for 89.4% of PCa cases and 91.2% of controls.

## 5. Conclusions

In conclusion, in this Spanish population-based case-control study, a slight protective association was found between compliance with the 2018 World Cancer Research Fund/American Institute for Cancer Research (WCRC/AICR) cancer prevention recommendations on diet, physical activity, and body fatness and overall PCa, especially in low aggressiveness PCa. In addition, limiting consumption of “fast foods” and other processed foods high in fat, starches or sugars, sugar-sweetened drinks, and alcohol were independently associated with lower risk of PCa. Altogether, it is advisable to comply with healthy lifestyles in the prevention of PCa.

## Figures and Tables

**Table 1:** The 2018 WCRF/AICR score construction in the CAPLIFE study.

Components	Personal Recommendations (Goals)	Operationalization	Scoring
1. Have a healthy weight	Ensure that body weight during childhood and adolescence projects toward the lower end of the healthy adult BMI range	*No information for operationalization*	n.a
	Keep your weight as low as you can within the healthy range throughout life	BMI ≥ 18.5–< 25 kg/m^2^	0.5
		BMI ≥ 25–< 30 kg/m^2^	0.25
		BMI ≥ 30 kg/m^2^ or < 18.5–25 kg/m^2^	0
	Avoid weight gain (measured as body weight or waist circumference) throughout adulthood	WC < 94 cm	0.5
		WC ≥ 94–<102 cm	0.25
		WC ≥ 102 cm	0
2. Be physically active	Be at least moderately physically active, and follow or exceed national guidelines	Total moderate-vigorous PA ≥ 150 min/week	1
		Total moderate-vigorous PA ≥ 75–< 150 min/week	0.5
		Total moderate-vigorous PA < 75 min/week	0
	Limit sedentary habits	*No information for operationalization*	n.a
3. Eat a diet rich in whole grains, vegetables, fruit, and beans	Consume a diet that provides at least 30 g/day of fiber from food sources	Dietary fiber intake ≥ 30 g/day	0.5
		Dietary fiber intake ≥ 15–<30 g/day	0.25
		Dietary fiber intake < 15 g/day	0
	Include more foods containing wholegrains, non-starchy vegetables, fruit, and pulses (legumes) such as beans and lentils	*No information for operationalization*	n.a.
	Eat a diet high in all types of plant foods including at least five portions or servings (at least 400 g or 15 oz in total) of a variety of non-starchy vegetables and fruit every day	Fruits and vegetables intake ^a^ ≥ 400 g/day	0.5
		Fruits and vegetables intake ^a^ ≥ 200–< 400 g/day	0.25
		Fruits and vegetables intake ^a^ < 200 g/day	0
	If you eat starchy roots and tubers as staple foods, eat non-starchy vegetables, fruit, and pulses (legumes) regularly too if possible	*No information for operationalization*	n.a.
4. Limit consumption of ”fast foods” and other processed foods high in fat, starches, or sugars	Limit consumption of processed foods high in fat, starches or sugars including “fast foods”, many prepared dishes, snacks, bakery foods and desserts, and confectionery (candy)	Percent of total kcal from ultra-processed foods (aUPFs): Tercile 1	1
		Tercile 2	0.5
		Tercile 3	0
5. Limit consumption of red and processed meat	If you eat red meat, limit consumption to no more than about three portions per week. Three portions are equivalent to about 350 to 500 g (about 12 to 18 oz) cooked weight of red meat. Consume very little, if any, processed meat.	Red meat <500 g/wk and processed meat < 21 g/wk	1
		Red meat <500 g/wk and processed meat ≥ 21–<100 g/wk	0.5
		Red meat ≥500 g/wk or processed meat ≥ 100g/wk	0
6. Limit consumption of sugar-sweetened drinks	Do not consume sugar-sweetened drinks	Sugary drinks intake ^b^ = 0 g/day	1
		Sugary drinks intake^b^ >0–≤ 250 g/day	0.5
		Sugary drinks intake ^b^ > 250 g/day	0
7. Limit alcohol consumption	For cancer prevention, it is best not to drink alcohol	No ethanol intake = 0 g/day	1
		Current ethanol intake > 0–< 28 g/day	0.5
		Current ethanol intake ≥ 28 g/day	0
8. Do not use supplements for cancer prevention	High-dose dietary supplements are not recommended for cancer prevention-aim to meet nutritional needs through diet alone	*No information for operationalization*	n.a
9. For mothers: breastfeed your baby, if you can	This recommendation aligns with the advice of the World Health Organization, which recommends infants are exclusively breastfed to 6 months, and then up to 2 years of age or beyond alongside appropriate complementary foods.	*Not applicable to this population*	n.a.
10. After a cancer diagnosis: follow our recommendations if you can	All cancer survivors should receive nutritional care and guidance on physical activity from trained professionals.	*Not applicable to this study*	n.a.
	Unless otherwise advised, and if you can, all cancer survivors are advised to follow the Cancer Prevention Recommendations as far as possible after the acute stage of treatment	*Not applicable to this study*	n.a.

Table made from the information provided by Shams-White MM et al. 2019 [16] AICR, American Institute for Cancer Research; BMI, body mass index; PA, physical activity; WC, waist circumference; WCRF, World Cancer Research Fund. ^a^ Fruits and vegetables excluding starchy vegetables (sweet potatoes), canned fruit, dried fruit, and fruit juices. ^b^ Sugary drinks included both sugar-sweetened soft-drinks and commercial fruit and vegetable juices.

**Table 2:** Characteristics of controls and prostate cancer (PCa) cases in CAPLIFE study.

	Controls	PCa Cases	*p*-Value
	(*n* = 302)	(*n* = 398)	
**Age (years), mean (SD)**	65.3 (8.2)	67.7 (7.4)	<0.01
Age (years), *n* (%)			<0.01
40–54	39 (12.9)	21 (5.3)	
55–69	162 (53.7)	217 (54.5)	
70–80	101 (33.4)	160 (40.2)	
Education, *n* (%)			0.38
Primary	92 (30.4)	119 (29.9)	
Secondary	147 (48.7)	211 (53.0)	
University	63 (20.9)	68 (17.1)	
Energy intake (kcal/day), mean (SD)	2438.1 (705.7)	2511.8 (617.6)	0.14
Alcohol consumption ^1^, *n* (%)			0.05
No consumption	56 (18.5)	69 (17.4)	
Moderate	215 (71.2)	262 (65.8)	
High	31 (10.3)	67 (16.8)	
Smoking status, *n* (%)			0.70
Never	88 (29.1)	105 (26.4)	
Former	157 (52.0)	212 (53.3)	
Current	57 (18.9)	81 (20.3)	
First-degree family history of PCa ^2^, *n* (%)			0.46
No	287 (95.0)	373 (93.7)	
Yes	15 (5.0)	25 (6.3)	
Aggressiveness, *n* (%)			
ISUP 1–2	–	307 (77.1)	
ISUP 3–5	–	91 (22.9)	
WCRF/AICR score, mean (SD) (Minimum–Maximum)	3.42 (1.01) (1.00–6.25)	3.27 (0.93) (0.75–6.25)	0.05
WCRF/AICR score, *n* (%)			0.08
≤1	1 (0.3)	3 (0.7)	
>1–≤2	27 (8.9)	30 (7.6)	
>2–≤3	98 (32.4)	160 (40.2)	
>3–≤4	105 (34.8)	132 (33.2)	
>4–≤5	51 (16.9)	63 (15.8)	
>5–≤6	18 (6.0)	8 (2.0)	
>6–≤7	2 (0.7)	1 (0.5)	
WCRF/AICR score ^3^, *n* (%)			0.69
Minimum compliance (T1)	101 (33.4)	140 (35.2)	
Intermediate compliance (T2)	111 (36.8)	151 (37.9)	
Maximum compliance (T3)	90 (29.8)	107 (26.9)	

SD, standard deviation. ^1^ Categories based on 2018 WCRF/AICR recommendations on alcohol consumption (g/day). ^2^ First-degree history of PCa in father and/or brothers. ^3^ Minimum compliance (Tercile 1: 0.75–2.75 points); intermediate compliance (Tercile 2: 3.00–3.75) and maximum compliance (Tercile 3: 4.00–6.25).

**Table 3:** Characteristics of participants in the control group according to terciles of the 2018 WCRF/AICR score in CAPLIFE study.

	Tercile 1 *n* = 101	Tercile 2 *n* = 111	Tercile 3 *n* = 90	*p*-Value
	min–max 0.75–2.75	min–max 3.00–3.75	min–max 4.00–6.50	
**Age (years), mean (SD)**	62.7 (7.8)	65.9 (7.3)	67.3 (8.9)	0.01
Age (years), *n* (%)				0.01
40–54	18 (17.8)	9 (8.1)	12 (13.3)	
55–69	62 (61.4)	64 (57.7)	36 (40.0)	
70–80	21 (20.8)	38 (34.2)	42 (46.7)	
Education, *n* (%)				0.74
Primary	30 (39.7)	35 (31.5)	27 (30.0)	
Secondary	54 (53.5)	51 (46.0)	42 (46.7)	
University	17 (16.8)	25 (22.5)	21 (23.3)	
Energy intake (kcal/day), mean (SD)	2510.5 (720.0)	2557.0 (685.3)	2210.3 (668.4)	<0.01
Alcohol consumption ^1^, *n* (%)				<0.01
No consumption	10 (9.9)	18 (16.2)	28 (31.1)	
Moderate	71 (70.3)	85 (76.6)	59 (65.6)	
High	20 (19.8)	8 (7.2)	3 (3.3)	
Smoking status, *n* (%)				0.26
Never	22 (21.8)	37 (33.3)	29 (32.2)	
Former	57 (56.4)	52 (46.9)	48 (53.3)	
Current	22 (21.8)	22 (19.8)	13 (14.4)	
First-degree history of PCa ^2^, *n* (%)				0.12
No	98 (97.0)	107 (96.4)	82 (91.1)	
Yes	3 (3.0)	3 (3.6)	8 (8.9)	

SD, standard deviation. ^1^ Categories based on WCRF/AICR recommendations on alcohol consumption (g/day). ^2^ First-degree history of PCa in father and/or brothers.

**Table 4:** Association between 2018 WCRF/AICR score and PCa stratified by aggressiveness in CAPLIFE study.

		WCRF/AICR Score Categories					
	N Control/Cases ^1^	Tercile 1	Tercile 2	Tercile 3	*p*-Trend	1-Unit Increase	*p*-Value
		aOR ^2^ (95% CI)	aOR ^2^ (95% CI)	aOR ^2^ (95% CI)		aOR ^2^ (95% CI)	
**Overall**							
	302/398	1.00	0.88 (0.61–1.27)	0.79 (0.53–1.19)	0.26	0.81 (0.69–0.96)	0.02
**Aggressiveness** **^2^**							
ISUP 1–2	302/307	1.00	0.87 (0.59–1.28)	0.73 (0.48–1.13)	0.16	0.79 (0.66–0.95)	0.01
ISUP 3–5	302/91	1.00	0.84 (0.47–1.53)	0.87 (0.46–1.66)	0.67	0.86 (0.69–1.06)	0.16

^1^ N, number; 95% CI, 95% confidence interval. ^2^ aOR: Odds ratio adjusted for age, educational level, smoking status, First-degree family history of PCa and total energy intake.

**Table 5:** Mutually adjusted odds ratios and 95% confidence intervals for PCa associated with the components of the 2018 WCRF/AICR score in CAPLIFE study.

Components of the Score	N Controls/Cases	aOR (95% CI) ^1^	*p*-Trend
1. Be a healthy weight ^2^			0.81
0–0.25	129/181	1	
0.5	85/87	0.69 (0.46–1.02)	
0.75–1	88/130	1.09 (0.75–1.60)	
2. Be physical active			0.63
0	227/315	1	
0.5	17/14	0.55 (0.26–1.19)	
1	58/69	0.94 (0.62–1.44)	
3. Eat a diet rich in whole grains, vegetables, fruit and beans ^2^			0.89
0–0.25	14/15	1	
0.5	16/26	1.35 (0.48–3.76)	
0.75–1	272/357	1.08 (0.47–2.50)	
4. Limit consumption of ‘fast foods’ and other processed foods high in fat, starches or sugars			0.02
0	101/163	1	
0.5	100/125	0.75 (0.51–1.10)	
1	101/110	0.63 (0.42–0.94)	
5. Limit consumption of red and processed meat			0.22
0	231/297	1	
0.5	65/82	0.96 (0.63–1.46)	
1	6/19	2.82 (1.05–7.59)	
6. Limit consumption of sugar-sweetened drinks			0.02
0	7/25	1	
0.5	132/179	0.39 (0.16–0.97)	
1	163/194	0.30 (0.12–0.77)	
7. Limit alcohol consumption			0.03
0	31/67	1	
0.5	215/262	0.57 (0.34–0.95)	
1	56/69	0.49 (0.27–0.91)	

N, number; 95% CI, 95% confidence interval. ^1^ aOR, Odds ratio adjusted for each individual component of the score and potential confounders (age, educational level, smoking status, primary family history of PCa and total energy intake). ^2^ For recommendations based on subitem, the possible scores could be: 0, 0.25, 0.5, 0.75 and 1, which were recategorized at 0–0.25, 0.5 and 0.75–1.

## Supplementary Materials

The following are available online at https://www.mdpi.com/2072-6643/12/3/768/s1. Table S1: Characteristics of controls and PCa cases with and without WCRF/AICR score in CAPLIFE study. Table S2: Mutually adjusted odds ratios and 95% confidence intervals for low and high aggressiveness PCa associated with the components of the 2018 WCRF/AICR score in CAPLIFE study.
